# Antimicrobial activity of copper-nickel coated door handles: a blinded, randomized controlled study in a clinical setting

**DOI:** 10.3205/dgkh000576

**Published:** 2025-08-20

**Authors:** Evgeny A. Idelevich, Andreas Schlattmann, Cristina Sauerland, Carsten Gebert, Karsten Becker

**Affiliations:** 1 Friedrich Loeffler-Institute of Medical Microbiology, University Medicine Greifswald, Greifswald, Germany; 2 Institute of Medical Microbiology, University Hospital Münster, Münster, Germany; 3 Institute of Biostatistics and Clinical Research, University of Münster, Münster, Germany; 4 Department of Tumor and Revision Surgery, Orthopedic Hospital Volmarstein, Wetter, Germany

**Keywords:** copper, antimicrobial activity, door handle, clinical setting, randomized controlled study

## Abstract

**Background:**

Prevention of nosocomial infections continues to be crucial to ensure patient safety and improve healthcare outcomes. In this regard, surface contamination plays an important role in the undetected transmission of nosocomial pathogens as a continuous, sporadic event or in the context of outbreaks. However, the impact of reducing bacterial contamination through copper-coated surfaces remains controversial.

**Methods:**

A pilot study was set up in a blinded, randomized controlled design to elucidate the antimicrobial activity of door handles coated with a copper-nickel alloy. Twelve doors in a specialized department of tumor and revision surgery of a German orthopedic hospital were randomly selected to install visually indistinguishable stainless-steel door handles, either without coating (control group, n=6) or with an alloy coating consisting of 30% copper and 70% nickel (study group, n=6). Patients, all involved personnel and investigators were blinded with regard to the assignment of door handles. Door handles were sampled for viable microorganisms at 24 h after disinfection by (i) consecutive use of wet and dry swabs and (ii) contact agar slides. Bacterial growth was detected and bacteria were identified using MALDI-TOF mass spectrometry. In addition, contamination kinetics of door handles were determined by ATP measurement at time points 0 h, 1 h, 2 h, 4 h, 8 h, 12 h and 24 h after disinfection. Each technique was used on three subsequent days.

**Results:**

Using swab method, the mean total number of colony forming units (cfu) of control and copper-nickel alloy surfaces was 2.14 cfu/cm^2^ and 0.67 cfu/cm^2^, respectively, yielding a difference of 68.7% (p=0.27). Bacterial counts from contact agar slide samples resulted in 0.86 cfu/cm^2^ on control and 0.6 cfu/cm^2^ on coated door handles which equals a difference of 30.2% (p=0.31). ATP bioluminescence measured over three subsequent days from coated door handles showed a decreased bioburden by 70.8%, 23.1%, 55.5%, 79.7%, 45.9%, 56.0%, and 68.3% of relative light units compared to control door handles at time points 0 h (before disinfection), 0 h (after disinfection), 1 h, 2 h, 4 h, 8 h, and 12 h, respectively. Statistically significant differences (p<0.05) were obtained for time points 4 h and 12 h.

**Conclusion:**

Our data indicate a trend of reduced bacterial and overall bioburden on copper-nickel-coated door handles. Further, larger randomized controlled trials are warranted to investigate the influence of copper-coated surfaces on the prevention of hospital-acquired infections.

## Zusammenfassung

**Hintergrund:**

Die Prävention nosokomialer Infektionen ist nach wie vor von entscheidender Bedeutung zur Gewährleistung von Patientensicherheit und Gesundheitsversorgung. Hierbei spielen Oberflächenkontaminationen eine wichtige Rolle bei der unentdeckten Übertragung nosokomialer Pathogene sowohl als wiederkehrendes sporadisches Ereignis als auch im Rahmen von Ausbrüchen. Der Einfluss der Verringerung der bakteriellen Kontamination von kupferbeschichteten Oberflächen auf die Prävention nosokomialer Infektionen ist jedoch umstritten.

**Methode:**

Zur Untersuchung der antimikrobiellen Aktivität von Türgriffen, die mit einer Kupfer-Nickel-Legierung beschichtet sind, wurde eine Pilotstudie in einem verblindeten, randomisiert- kontrollierten Design durchgeführt. Zwölf Türen in einer Fachabteilung für Tumor- und Revisionschirurgie eines deutschen orthopädischen Krankenhauses wurden nach dem Zufallsprinzip ausgewählt, um optisch nicht unterscheidbare Türgriffe aus Edelstahl entweder ohne Beschichtung (Kontrollgruppe, n=6) oder mit einer Legierungsbeschichtung aus 30% Kupfer und 70% Nickel (Studiengruppe, n=6) zu installieren. Die Patienten, das gesamte beteiligte Personal und die Prüfer waren hinsichtlich der Zuordnung der Türklinken verblindet. Die Türgriffe wurden 24 h nach der Desinfektion mittels konsekutiver Verwendung von nassen und trockenen Tupfern sowie Kontaktagar auf lebensfähige Mikroorganismen untersucht. Die Proben wurden auf bakterielles Wachstum untersucht und angezüchtete Mikroorganismen wurden mittels MALDI-TOF-Massenspektrometrie identifiziert. Darüber hinaus wurde die Kontaminationskinetik von Türgriffen durch ATP-Messung zu den Zeitpunkten 0 h, 1 h, 2 h, 4 h, 8 h, 12 h und 24 h nach Desinfektion bestimmt. Jedes Verfahren wurde an drei aufeinander folgenden Tagen angewandt.

**Ergebnisse:**

Unter Verwendung der Tupfer-Methode betrug die mittlere Gesamtzahl der koloniebildenden Einheiten (KBE) auf den Oberflächen der Kontroll-Türgriffe und der Türgriffe mit Kupfer-Nickel-Legierung 2,14 cfu/cm^2^ bzw. 0,67 cfu/cm^2^, was einer Reduktion um 68,7% entspricht (p=0,27). Die ermittelten KBE der Kontaktagarproben umfassten 0,86 cfu/cm^2^ auf den Türgriffen der Kontrollgruppe und 0,6 cfu/cm^2^ auf den beschichteten Türgriffen, was einer Differenz von 30,2% entspricht (p=0,31). Die ATP-Biolumineszenz, die über drei aufeinanderfolgende Tage an den beschichteten Türgriffen gemessen wurde, zeigte eine Verringerung der bakteriellen Belastung um 70,8%, 23,1%, 55,5%, 79,7%, 45,9%, 56,0% und 68,3% der relativen Lichteinheiten (RLU) im Vergleich zu den Kontrolltürgriffen zu den Zeitpunkten 0 h (vor der Desinfektion), 0 h (nach der Desinfektion), 1 h, 2 h, 4 h, 8 h und 12 h, wobei für die Zeitpunkte 4 h und 12 h signifikante Unterschiede (p<0,05) in den RLU ermittelt wurden.

**Schlussfolgerung:**

Kupfer-Nickel-beschichtete Türgriffe zeigten im Vergleich zur Kontrollgruppe eine Tendenz sowohl zur Verringerung der bakteriellen Belastung als auch der Gesamtbelastung mit organischem Material. Ob die reduzierte bakterielle Belastung auf den Türklinken zur Prävention nosokomialer Infektionen beiträgt, kann nur durch aufwendige epidemiologische Studien geklärt werden.

## Introduction

Healthcare-associated infections (HAIs) affect approximately 4.1 million acute care patients in the European Union alone annually resulting in about 37,000 deaths directly attributable to HCAIs [1]. These and comparable estimates in other countries reflect a high disease and socioeconomic burden. Strategies to combat HCAIs are therefore warranted, especially given the increase of antibiotic resistant pathogens. Heavy metals and metalloids such as arsenic, copper, manganese, mercury, silver and zinc have long been used as antimicrobial agents in human and veterinary medicine [2], [3].

One promising approach of decreasing bacterial burden in healthcare facilities is coating hard surfaces as well as bed linens and clothes with continuously active antimicrobial metals. Copper, as one of these self-sanitizing metals, has long been shown to possess strong antimicrobial activity and to be effective in reducing colonization of abiotic surfaces and HCAIs with resistant organisms in various hospital settings [4], [5], [6], [7], [8], [9], [10], [11]. Inactivation of microorganisms is also achieved by using copper alloys, depending on the percentage of copper in the alloy [5], [12], [13], [14]. In 2008, the U. S. Environmental Protection Agency (EPA) registered copper surfaces as antimicrobial material [15] and, recently, EPA announced that it has registered copper alloys that have shown effectiveness against viruses, including SARS-CoV-2 [16]. Otherwise, study results have also been reported that have not demonstrated a reduction in HCAIs with the use of copper [17] and a recent meta-analysis has found only low-quality evidence of potential clinical significance [18]. However, the studies were frequently limited by lack of randomization, incomplete blinding, weak study design and/or high risk of bias and larger clinical trials on the impact of copper treatment are requested [7], [17], [18], [19], [20], [21], [22], [23], [24], [25]. 

In this pilot study, we applied a blinded randomized design to assess the microbial burden and overall contamination of copper-nickel alloy-coated and control door handles in a hospital setting.

## Methods

### Study design 

The study was performed at the Department of Tumor and Revision Surgery in the Orthopaedic Hospital Volmarstein, Wetter, Germany. The investigators, hospital staff and patients were blinded regarding the assignment of coated and control door handles. As control for the efficacy of the applied disinfectant (Terralin^®^ protect-solution, Schülke & May, Norderstedt, Germany), all study door handles (Häfele, Nagold, Germany) were disinfected on the first day of each study week and sampled after the required exposure time had elapsed. Study samples were taken three days in a row at the same time of day. After each swab and contact slide sample acquisition as well as after the last ATP measurement, the door handles were disinfected according to the local hygiene plan. No further cleaning or disinfection of the door handles included in this study was executed by maintenance and hospital staff during the study interval.

To avoid differences in behavior of patients, visitors and hospital staff, and to prevent biases introduced by the investigators, we used the stainless-steel door handles with the copper-nickel cation coating (n=6) (Alasept, Häfele, Nagold, Germany) which does not change the visible appearance of the door handle; therefore, treatment and control stainless-steel door handles without coating (n=6) were visually indistinguishable. Three different methods were applied to determine the environmental burden of the door handles: Swabbing with subsequent cultivation, direct sampling using contact slides, and ATP bioluminescence measurements which detect ATP-containing organic residues.

### Double swab technique 

Two Quick Swabs (3M, Neuss, Germany) were used to sample each door handle. The first sample was taken with a wet swab, i.e. after the neutralizing buffer-containing cap was snapped off and had soaked the material. Then, the swab was streaked horizontally over the door handle multiple times while constantly being rotated. The remaining surface was sampled by vertically moving the swab in a meandering pattern along the door handle and afterwards placed it back into its sample tube. The procedure was repeated with the second, dry swab; after being placed back into its sample tube, the buffer-containing cap was torn off and the material soaked.

The sample tubes were vortexed for two minutes. Physiological saline solution was used to prepare 1:10, 1:100, and 1:1000 dilutions of the buffer of the original sample in a total volume of 1 mL. One blood, three brain-heart-infusion (BHI) and one Schaedler agar plate (all BD, Heidelberg, Germany) were inoculated with 100 µL of the original sample and its dilutions. Incubation temperature for all plates was 37°C. The Schaedler agar plate was incubated in an anaerobic jar using AnaeroGen sachets (Oxoid Deutschland, Wesel, Germany) and examined for a first colony count after 72 h; first colony counts for the blood and BHI agar plates were performed after 24 h. A final colony count on all plates was performed after five days of incubation. Bacterial colonies were isolated, identified using matrix-assisted laser desorption/ionization time-of-flight mass spectrometry (MALDI-TOF MS; Bruker Daltonics, Bremen, Germany) and frozen at –80°C.

### Contact slide method 

Two HYCON^®^ contact slides (Merck KGaA, Darmstadt, Germany) were used including a tryptic soy agar for determination of total bacterial count (type TC) and a sheep blood supplemented agar for fastidious bacteria (type B). Contact slides were consecutively placed on the top left (type TC) and top right (type B) side of the door handle, wrapped around it and removed. They were placed back into their respective boxes and sealed for transport. The contact slides were incubated for 24 h at 37°C before the first colony count; after four more days, a final colony count was performed and bacterial colonies were isolated. Isolates were frozen at –80°C after identification by MALDI-TOF MS.

### ATP-bioluminescence assay 

Samples for bioluminescence measurements using the Clean-Trace™ NG luminometer were taken with Clean-Trace™ surface swabs (both 3M Deutschland GmbH, Neuss, Germany) at time points 0 h (before disinfection), 0 h (after disinfection), 1 h, 2 h, 4 h, 8 h, and 12 h. The measurements were performed according to the manufacturer’s instructions. To avoid the impact of sampling-induced “cleaning” effects on the results, the surface of each door handle was divided into six identical areas. These were sampled in a randomly allocated sequential order. The whole door handle was sampled at 0 h before disinfection.

### Statistical evaluation 

Generalized estimating equations (GEE) including Wald statistics were used to control for the clustering of bacterial counts within door handles type over the three-day sampling time and to analyze a possible relationship between allocation or sampling day [26].

## Results

### Double swab technique 

The results for the double swab technique yielded values ranging from 0 cfu/cm^2^ to 27.25 cfu/cm^2^ with a mean of 2.14 cfu/cm^2^ (median 0.56 cfu/cm^2^, standard deviation 3.56 cfu/cm^2^) for the uncoated door handles.

The coated door handles tested with the double swab technique were found to be colonized by 0.02 cfu/cm^2^ to 3.98 cfu/cm^2^ with a mean of 0.67 cfu/cm^2^ (median 0.56 cfu/cm^2^, standard deviation 0.54 cfu/cm^2^). The differences did not reach statistical significance (p=0.27).

**Figure 1:**
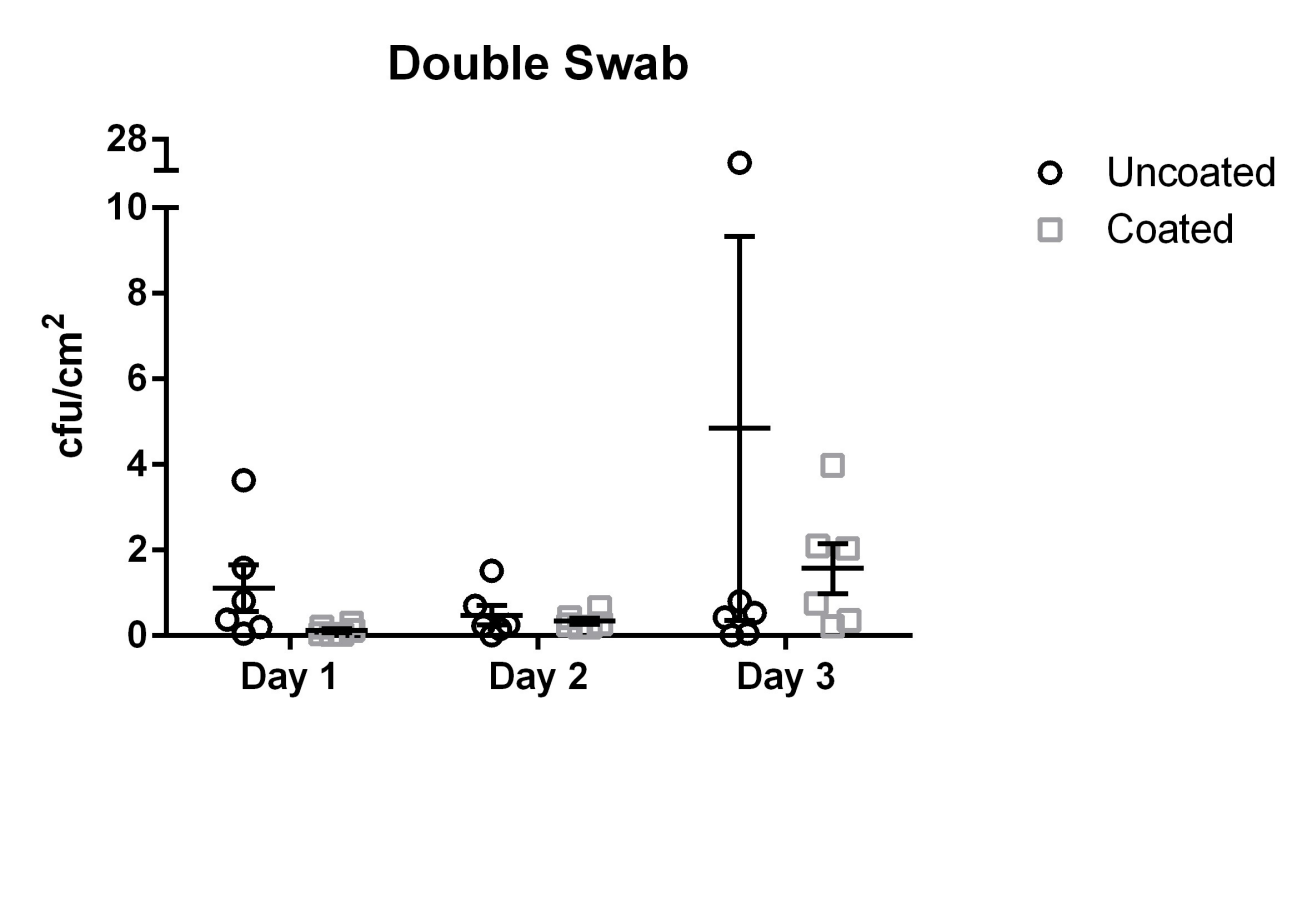
Means and standard error of the means of microbial load (cfu/cm^2^) found using the double swab technique. Samples were taken on three subsequent days from six uncoated (black circles) and six copper-nickel-coated (gray squares) door handles.

The means and standard error of the means (SEM) of uncoated and coated door handles examined with the double swab technique are shown in Figure 1 (Fig. 1).

### Contact slide method 

The results for the contact slide method yielded results ranging from 0.04 cfu/cm^2^ to 3.3 cfu/cm^2^ with a mean of 0.86 cfu/cm^2^ (median 0.77 cfu/cm^2^, standard deviation 0.59 cfu/cm^2^) for the uncoated door handles.

**Figure 2:**
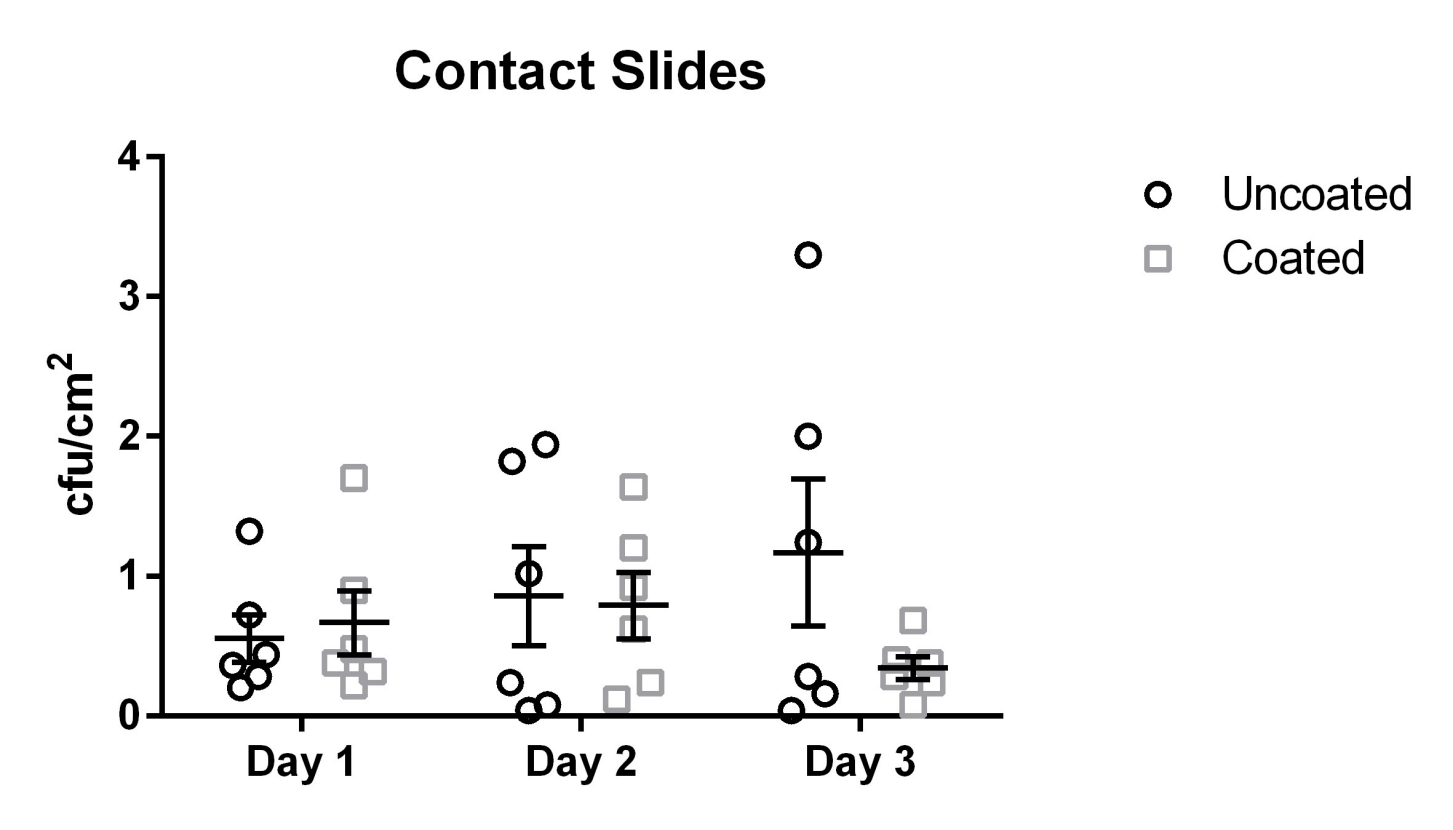
Means and standard error of the means of microbial load (cfu/cm^2^) found using the contact slide method. Samples were taken on three subsequent days from six uncoated (black circles) and six copper-nickel-coated (gray squares) door handles.

The coated door handles tested with the contact slide method were colonized by 0.01 cfu/cm^2^ to 1.7 cfu/cm^2^ with a mean of 0.6 cfu/cm^2^ (median 0.47 cfu/cm^2^, standard deviation 0.34 cfu/cm^2^) (Figure 2 (Fig. 2)). The differences did not reach statistical significance (p=0.31).

### ATP-bioluminescence assay 

The measurements for the ATP-bioluminescence assay for the uncoated door handles ranged from 5 relative light units (RLU) to 33,760 RLU (day 1), 30 RLU to 24,984 RLU (day 2), and 114 RLU to 4,875 RLU (day 3); measurements from coated door handles ranged from 11 RLU to 1,684 RLU (day 1), 29 RLU to 7,111 RLU (day 2), and 91 RLU to 3,173 RLU (day 3). 

ATP bioluminescence measured over three subsequent days from coated door handles showed a decreased bioburden by 70.8% (p=0.13), 23.1% (p=0.23), 55.5% (p=0.05), 79.7% (p=0.19), 45.9% (p=0.03), 56.0% (p=0.18), and 68.3% (p=0.004) of relative light units compared to control door handles at time points 0 h (before disinfection), 0 h (after disinfection), 1 h, 2 h, 4 h, 8 h, and 12 h, respectively. Hence, statistically significant differences (p<0.05) were obtained for time points 4 h and 12 h. 

**Figure 3:**
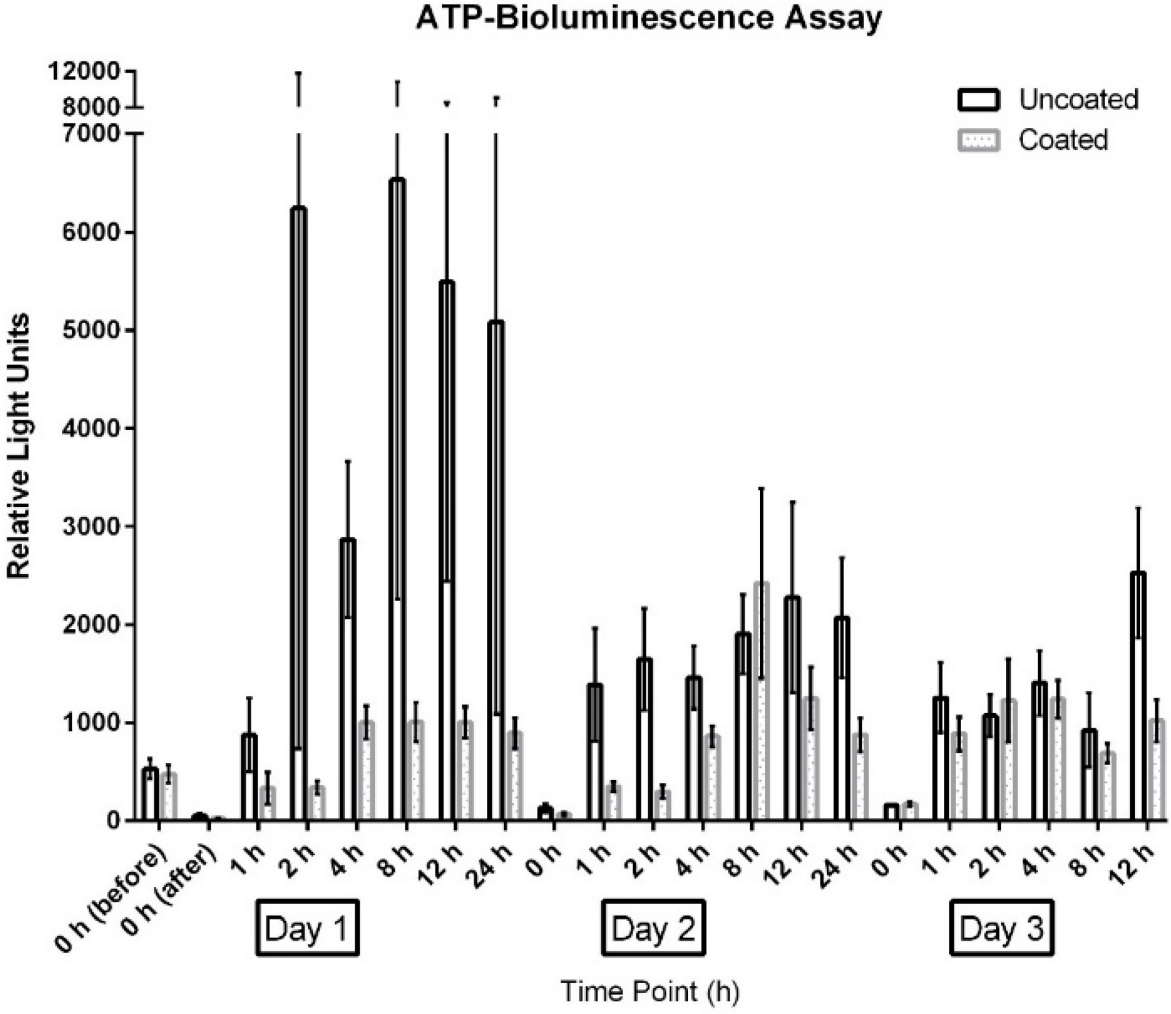
Means and standard error of the means of ATP-bioluminescence measurements over three days. Samples were taken from six uncoated (black) and six copper-nickel-coated (gray) door handles. “before”: before disinfection; “after”: after disinfection.

**Figure 4:**
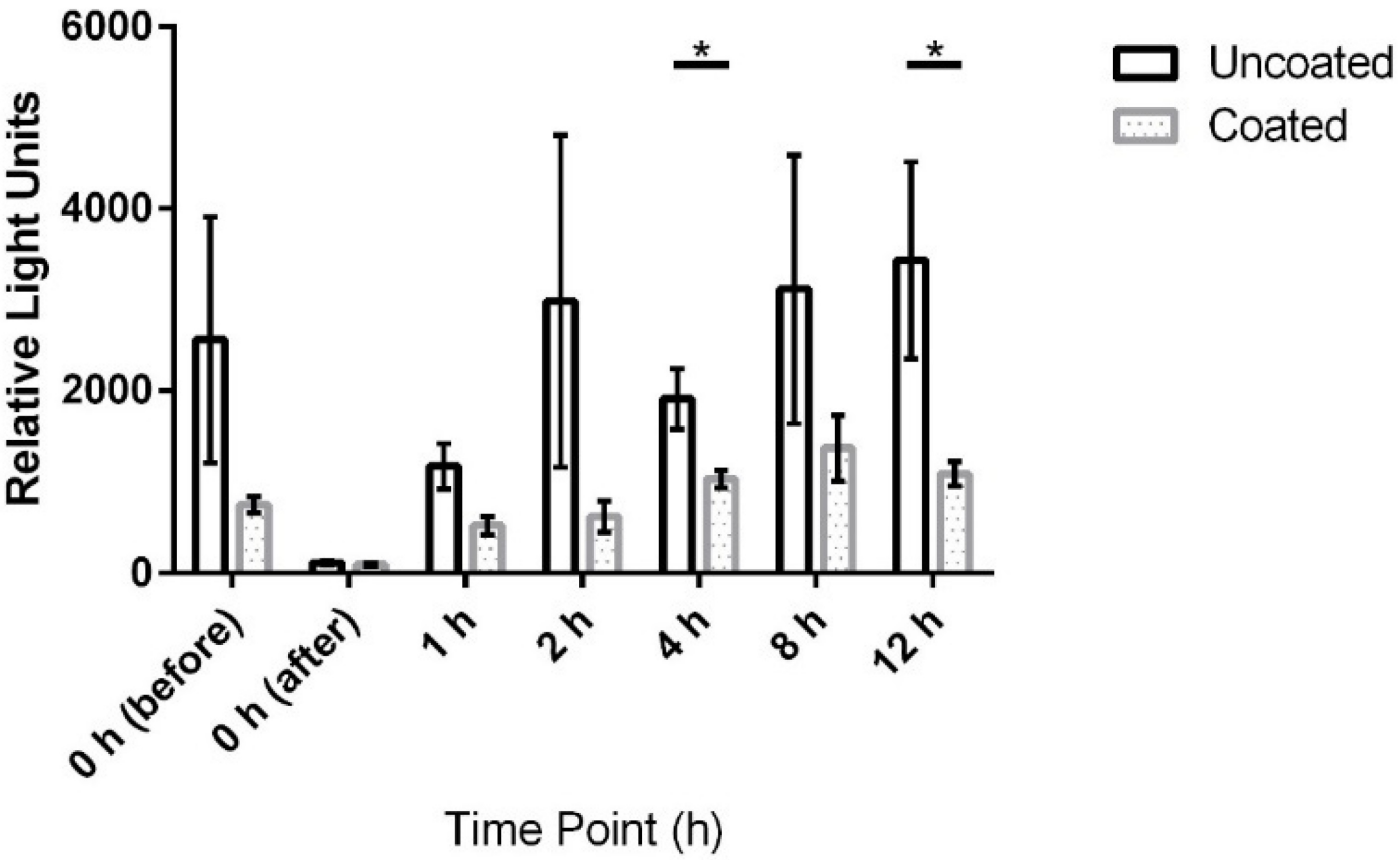
Means and standard error of the means of ATP-bioluminescence measurements at measured time points over three days. Samples were taken from six uncoated (black) and six copper-nickel-coated (gray) door handles. Statistically significant differences (p<0.05) were found for time points 4 h and 12 h. “before”: before disinfection; “after”: after disinfection.

**Figure 5:**
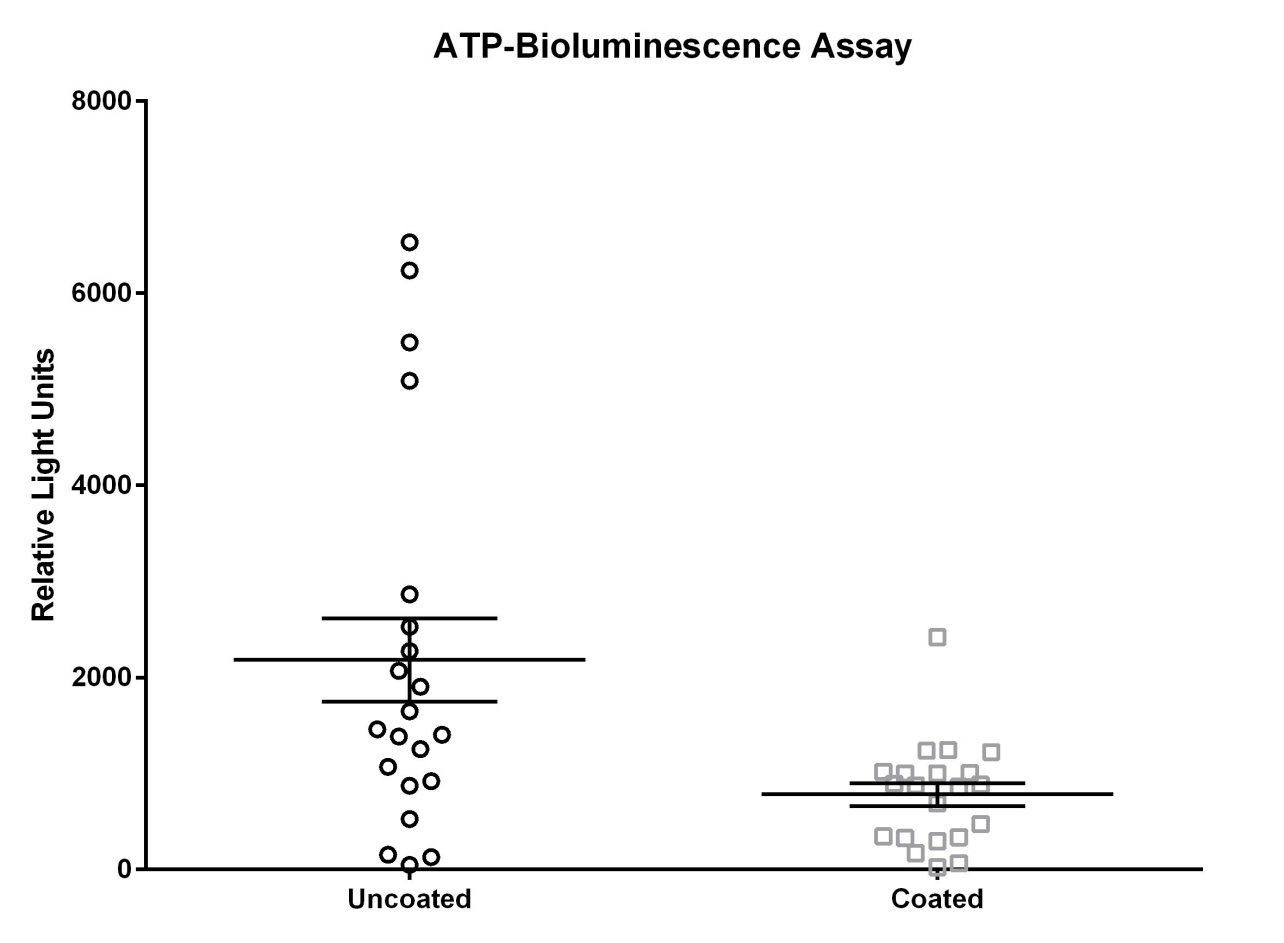
Means and standard error of the means of ATP-bioluminescence measurements over three days. Samples were taken from six uncoated (black) and six copper-nickel-coated (gray) door handles.

The results for uncoated and coated door handles examined with the ATP-bioluminescence assay are shown in Figure 3 (Fig. 3) and Figure 4 (Fig. 4); the former depicts every time point measured, the latter shows the summary of all time points. In Figure 5 (Fig. 5), all measurements from uncoated and coated door handles are shown.

### Identification by MALDI-TOF MS 

**Figure 6:**
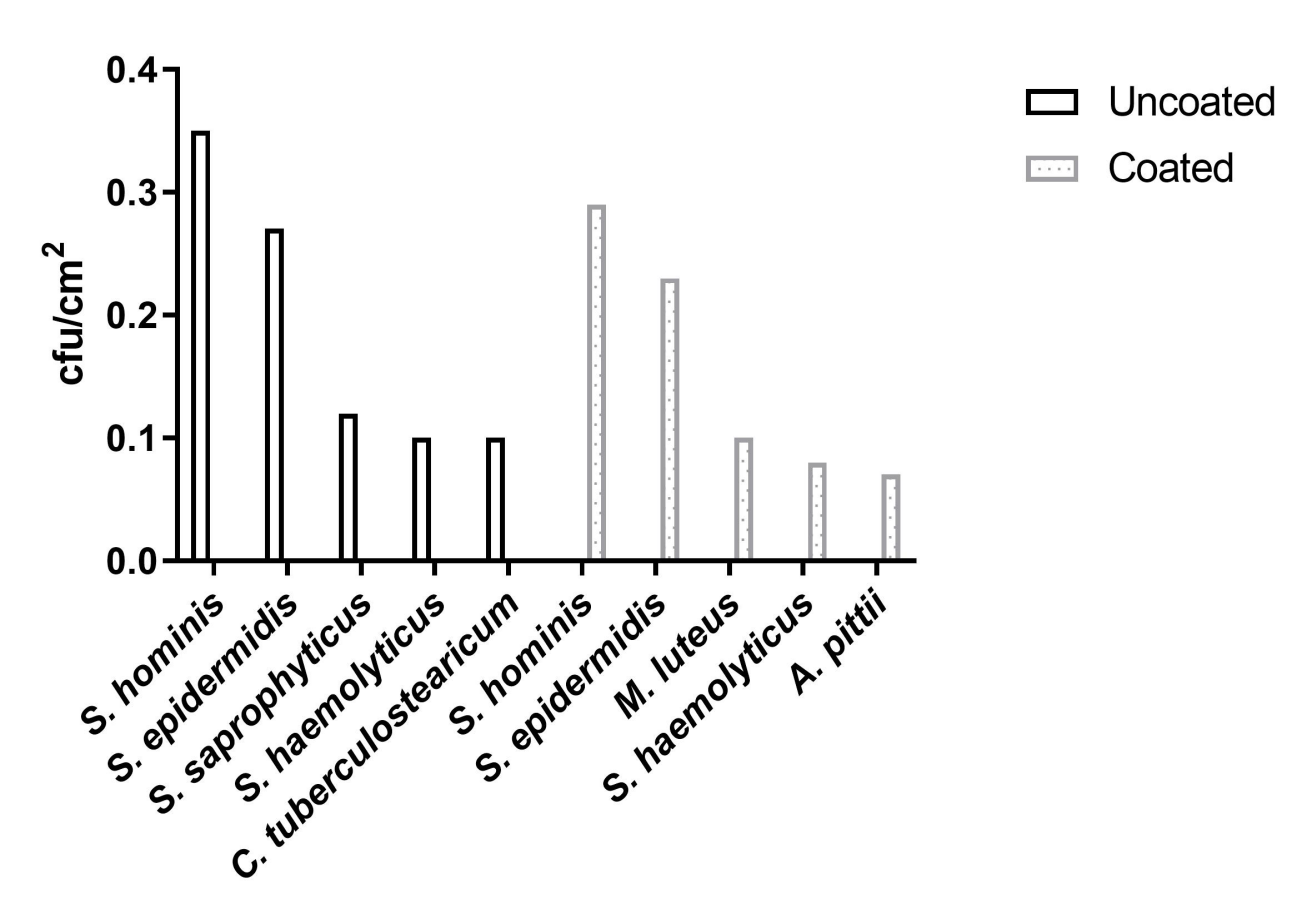
Most abundant bacterial species isolated by contact slide method and double swab technique from coated and uncoated door handles.

Using MALDI-TOF MS, the cultured bacteria from the double swab technique and the contact slide method were identified: *Staphylococcus hominis* and *S. epidermidis* were most abundant species isolated from both coated and uncoated door handles (0.29 vs. 0.35 cfu/cm^2^ and 0.23 vs. 0.27 cfu/cm^2^, respectively), followed by *Micrococcus luteus* (0.10 cfu/cm^2^), *S. haemolyticus* (0.08 cfu/cm^2^), and *Acinetobacter pittii* (0.07 cfu/cm^2^) for the coated door handles and *S. saprophyticus* (0.12 cfu/cm^2^), *S. haemolyticus* (0.10 cfu/cm^2^) and *Corynebacterium* *tuberculostearicum* (0.10 cfu/cm^2^) for the uncoated door handles (Figure 6 (Fig. 6)).

## Discussion

Heavy metals and metalloids as well as the technological advancement towards metal and metal oxide-based nanoparticles for better delivery of the metals are known for their non-specific antimicrobial activities with a broad spectrum of activity, which fosters their use as antimicrobial substrates for various medical applications [27], [28], [29]. This study used a blinded, randomized controlled design to investigate the impact of copper surfaces in a clinical setting. The data indicate an effect on the overall colonization and organic burden of door handles depending on the surface material. This is in agreement with most publications to date that assessed microbial burden. There are some aspects, however, in which our findings differ from previously published data. Salgado and colleagues, for instance, found that the cumulative burden in rooms with copper-surfaced objects showed a 0.76 log (about 5.75-fold) reduction compared to rooms without these objects [7]; and while our data show a trend of decreased bacterial colonization, we did not observe a reduction of this magnitude. The findings of Casey et al. whose copper-containing items harbored between 90% and 100% fewer microorganisms are closer to our results [4] but they report that 5/10 control sample points and none of the copper points failed the benchmark values for hand contact surfaces of a total aerobic colony count of <5 cfu/cm^2^ [30], [31]. In our study, this target was failed only once by a control door handle.

These discrepancies can have several reasons: One explanation could be that most of the previous studies did not include door handles, although they examined frequently touched surfaces in hospitals and patient rooms. Additionally, the coating used in the present study was an alloy of 30% copper and 70% nickel. In the USA, however, the copper alloy group with the lowest copper percentage registered with the Environmental Protection Agency (EPA) is 60%. The comparatively low contamination of control door handles could be due to the setting which is in this case an orthopedic ward and not an acute medical ward or intensive care unit as is the case in many other studies performed in hospital settings [4], [5], [6], [7]. The low contamination of the door handles in the control group also indicates very good implementation of hand hygiene measures by medical staff and patients as well as of disinfection measures and underlines their importance [32], [33].

In the meantime, numerous studies have underlined the antibacterial effect of copper surfaces, including against multidrug-resistant organisms (MDROs), in the clinical environment [34], [35], [36]. Also other strategies in surface chemistry have been proposed such as hydrogel-coated antibacterial surfaces and they have been recognized as an effective strategy to reduce bacterial colonization [37]. As an example, respective hydrogels were developed combining the superhydrophilic and antifouling nature of an polysulfobetaine network with the antibacterial effects of copper ions [38].

The statistically significant differences at time points 4 h and 12 h obtained with the ATP-bioluminescence assay indicate accordance with findings from Mikolay et al. who found delayed recolonization of copper-containing alloys [39]. The relatively small sample size in this pilot study might be the reason why other time points did not show statistical significance but rather only a trend towards decreased bioburden. Notably, all three sampling methods used in this study, similarly indicated reduced bacterial or overall bioburden on coated door handles. 

While the data of other authors show that overall colonization and especially colonization with MDROs in intensive care units and other specialized wards is significantly decreased by using copper-coated surfaces and items, the findings of our study – despite its small sample size – indicate that the microbial burden in a standard ward in Germany can be well within limits even without copper-coated door handles. A recent study emphasized that the efficacy of copper surfaces is greatly influenced by the frequency of cleaning and disinfection, with the greatest benefit expected in areas where cleaning is challenging [14]. It should also be taken into account that exposure to copper can facilitate antibiotic resistance development [40], [41]. It is known for copper and other heavy metals (e.g. cadmium, chromium, zinc, and vanadium) as well as metalloids (arsenic) that the selection of antibiotic-resistant bacteria can be influenced by them through co-selective and cross-resistance processes [42], [43], [44]. Some of them are permitted as additives in animal feed to promote growth and combat diarrhea [45]. So it is worrying that copper tolerance has been described in bacteria from fresh produce [46], especially considering possible co-resistances with classical antibiotics. This has been shown, e.g., for copper resistance in enterococci associated with macrolide and glycopeptides resistance [47] and zinc resistance linked to staphylococcal methicillin resistance [48]. Possible heavy metal resistance of bacterial species could also be of importance in human medicine, as some medical products such as nasal sprays and antiseptic solutions contain heavy metals and implanted metallic medical devices release metals at the site of implantation [49]. Recent outbreaks with multidrug-resistant and hypervirulent *Klebsiella pneumoniae* lineages carrying several plasmid-encoded genes responsible for heavy metal resistance such as *ars* (arsenic/antimony resistance), *sil* (silver resistance), and *pco* (copper resistance) emphasize the importance of this problem [50].

### Limitations

Our study has some limitations; the most important of which is the small sample size. However, the number of installed coated and uncoated door handles reflects the size of the orthopedic unit in which the study was performed. As a result, the influence of random differences in the frequency of use of the individual door handles may have a particular effect. Compared to the size of other surfaces in a hospital environment, door handles only account for a small portion, so their influence on the HAI rate may generally appear low. However, as “high-touched surfaces”, door handles in hospitals constitute a surface that is heavily used by medical and technical staff, patients and visitors, and therefore require special attention as part of hygiene measures [51], [52]. In addition to reducing bacterial contamination through surface coating, non-contact door opening systems can also be a successful strategy for preventing pathogen transmission [53]. Our study only addressed a single aspect of the variety of measures to prevent nosocomial infections. However, it has been shown that multimodal strategies with bundled approaches are the best way to achieve a sustainable reduction in healthcare-associated infections [54], [55].

## Conclusions

Randomized controlled trials in clinical settings are needed to prove, whether the observed trend of reduced microbial burden on copper surfaces leads to a decrease of HAIs. Furthermore, studies investigating induced resistance to copper and antibiotics are warranted to elucidate and possibly exclude any undesired effects on MDRO selection.

## Notes

### Competing interests

The authors declare that they have no competing interests.

### Funding

The study was supported by internal institutional funds. The door handles were provided free of charge by Häfele GmbH & Co KG (Nagold, Germany). No other financial support was received for this study from third parties. 

Availability of data and materials

The datasets used and analyzed during the current study are available from the corresponding author on reasonable request.

### Acknowledgments

We are thankful to B. Grünastel, M. Tigges and I. Sielemann, as well as the staff of the Orthopedic Hospital Volmarstein, for support.

### Author’s ORCIDs

- Becker K: https://orcid.org/0000-0002-6391-1341
- Idelevich EA: https://orcid.org/0009-0009-4207-5290
- Sauerland C: https://orcid.org/0000-0002-8748-8218

## References

[1] European Centre for Disease Prevention and Control (2013). Point prevalence survey of healthcare-associated infections and antimicrobial use in European acute care hospitals.

[2] Frei A, Verderosa AD, Elliott AG, Zuegg J, Blaskovich MAT (2023). Metals to combat antimicrobial resistance. Nat Rev Chem.

[3] Turner RJ (2024). The good, the bad, and the ugly of metals as antimicrobials. Biometals.

[4] Casey AL, Adams D, Karpanen TJ, Lambert PA, Cookson BD, Nightingale P, Miruszenko L, Shillam R, Christian P, Elliott TS (2010). Role of copper in reducing hospital environment contamination. J Hosp Infect.

[5] Karpanen TJ, Casey AL, Lambert PA, Cookson BD, Nightingale P, Miruszenko L, Elliott TS (2012). The antimicrobial efficacy of copper alloy furnishing in the clinical environment: a crossover study. Infect Control Hosp Epidemiol.

[6] Schmidt MG, Attaway HH, Sharpe PA, John J, Sepkowitz KA, Morgan A, Fairey SE, Singh S, Steed LL, Cantey JR, Freeman KD, Michels HT, Salgado CD (2012). Sustained reduction of microbial burden on common hospital surfaces through introduction of copper. J Clin Microbiol.

[7] Salgado CD, Sepkowitz KA, John JF, Cantey JR, Attaway HH, Freeman KD, Sharpe PA, Michels HT, Schmidt MG (2013). Copper surfaces reduce the rate of healthcare-acquired infections in the intensive care unit. Infect Control Hosp Epidemiol.

[8] Lazary A, Weinberg I, Vatine JJ, Jefidoff A, Bardenstein R, Borkow G, Ohana N (2014). Reduction of healthcare-associated infections in a long-term care brain injury ward by replacing regular linens with biocidal copper oxide impregnated linens. Int J Infect Dis.

[9] Schmidt MG, Tuuri RE, Dharsee A, Attaway HH, Fairey SE, Borg KT, Salgado CD, Hirsch BE (2017). Antimicrobial copper alloys decreased bacteria on stethoscope surfaces. Am J Infect Control.

[10] Butler JP (2018). Effect of copper-impregnated composite bed linens and patient gowns on healthcare-associated infection rates in six hospitals. J Hosp Infect.

[11] Abraham J, Dowling K, Florentine S (2021). Can Copper Products and Surfaces Reduce the Spread of Infectious Microorganisms and Hospital-Acquired Infections? Materials (Basel).

[12] Niiyama N, Sasahara T, Mase H, Abe M, Saito H, Katsuoka K (2013). Use of copper alloy for preventing transmission of methicillin-resistant Staphylococcus aureus contamination in the dermatology ward. Acta Derm Venereol.

[13] Souli M, Antoniadou A, Katsarolis I, Mavrou I, Paramythiotou E, Papadomichelakis E, Drogari-Apiranthitou M, Panagea T, Giamarellou H, Petrikkos G, Armaganidis A (2017). Reduction of Environmental Contamination With Multidrug-Resistant Bacteria by Copper-Alloy Coating of Surfaces in a Highly Endemic Setting. Infect Control Hosp Epidemiol.

[14] Bryce EA, Velapatino B, Donnelly-Pierce T, Khorami HA, Wong T, Dixon R, Asselin E, McGeer A, Srigley JA, Katz K (2022). Antimicrobial efficacy and durability of copper formulations over one year of hospital use. Infect Control Hosp Epidemiol.

[15] Grass G, Rensing C, Solioz M (2011). Metallic copper as an antimicrobial surface. Appl Environ Microbiol.

[16] Chemical Safety and Pollution Prevention Headquarter of the U.S. Environmental Protection Agency (EPA) (2021). EPA Registers Copper Surfaces for Residual Use Against Coronavirus.

[17] Marik PE, Shankaran S, King L (2020). The effect of copper-oxide-treated soft and hard surfaces on the incidence of healthcare-associated infections: a two-phase study. J Hosp Infect.

[18] Albarqouni L, Byambasuren O, Clark J, Scott AM, Looke D, Glasziou P (2020). Does copper treatment of commonly touched surfaces reduce healthcare-acquired infections? A systematic review and meta-analysis. J Hosp Infect.

[19] Muller MP, MacDougall C, Lim M, Ontario Agency for Health Protection and Promotion Public Health Ontario, Provincial Infectious Diseases Advisory Committee on Infection Prevention and Control, Provincial Infectious Diseases Advisory Committee on Infection Prevention and Control (2016). Antimicrobial surfaces to prevent healthcare-associated infections: a systematic review. J Hosp Infect.

[20] Weber DJ, Otter JA, Rutala WA (2017). Can Copper-Coated Surfaces Prevent Healthcare-Associated Infections? Infect Control Hosp Epidemiol.

[21] Sifri CD, Burke GH, Enfield KB (2016). Reduced health care-associated infections in an acute care community hospital using a combination of self-disinfecting copper-impregnated composite hard surfaces and linens. Am J Infect Control.

[22] Marcus EL, Yosef H, Borkow G, Caine Y, Sasson A, Moses AE (2017). Reduction of health care-associated infection indicators by copper oxide-impregnated textiles: Crossover, double-blind controlled study in chronic ventilator-dependent patients. Am J Infect Control.

[23] Zerbib S, Vallet L, Muggeo A, de Champs C, Lefebvre A, Jolly D, Kanagaratnam L (2020). Copper for the Prevention of Outbreaks of Health Care-Associated Infections in a Long-term Care Facility for Older Adults. J Am Med Dir Assoc.

[24] von Dessauer B, Navarrete MS, Benadof D, Benavente C, Schmidt MG (2016). Potential effectiveness of copper surfaces in reducing health care-associated infection rates in a pediatric intensive and intermediate care unit: A nonrandomized controlled trial. Am J Infect Control.

[25] Rivero P, Brenner P, Nercelles P (2014). Impacto del cobre en la reducción de infecciones intrahospitalarias, mortalidad y gasto en antimicrobianos en una Unidad de Cuidados Intensivo de adultos. Rev Chilena Infectol.

[26] Liang KY, Zeger SL (1986). Longitudinal Data-Analysis Using Generalized Linear-Models. Biometrika.

[27] Reda AT, Park JY, Park YT (2024). Zinc Oxide-Based Nanomaterials for Microbiostatic Activities: A Review. J Funct Biomater.

[28] Jones N, Ray B, Ranjit KT, Manna AC (2008). Antibacterial activity of ZnO nanoparticle suspensions on a broad spectrum of microorganisms. FEMS Microbiol Lett.

[29] Paladini F, Pollini M, Sannino A, Ambrosio L (2015). Metal-Based Antibacterial Substrates for Biomedical Applications. Biomacromolecules.

[30] Dancer SJ (2004). How do we assess hospital cleaning? A proposal for microbiological standards for surface hygiene in hospitals. J Hosp Infect.

[31] White LF, Dancer SJ, Robertson C, McDonald J (2008). Are hygiene standards useful in assessing infection risk?. Am J Infect Control.

[32] Kramer A, Seifert J, Abele-Horn M, Arvand M, Biever P, Blacky A, Buerke M, Ciesek S, Chaberny I, Deja M, Engelhart S, Eschberger D, Gruber B, Hedtmann A, Heider J, Hoyme UB, Jäkel C, Kalbe P, Luckhaupt H, Novotny A, Papan C, Piechota H, Pitten FA, Reinecke V, Schilling D, Schulz-Schaeffer W, Sunderdiek U (2024). S2k-Guideline hand antisepsis and hand hygiene. GMS Hyg Infect Control.

[33] Boyce JM, Pittet D, Healthcare Infection Control Practices Advisory Committee, HICPAC/SHEA/APIC/IDSA Hand Hygiene Task Force (2002). Guideline for Hand Hygiene in Health-Care Settings. Recommendations of the Healthcare Infection Control Practices Advisory Committee and the HICPAC/SHEA/APIC/IDSA Hand Hygiene Task Force. Society for Healthcare Epidemiology of America/Association for Professionals in Infection Control/Infectious Diseases Society of America. MMWR Recomm Rep.

[34] Bondareva NE, Sheremet AB, Morgunova EY, Khisaeva IR, Parfenova AS, Chernukha MY, Omran FS, Emelyanenko AM, Boinovich LB (2024). Study of the Antibacterial Activity of Superhydrophilic and Superhydrophobic Copper Substrates against Multi-Drug-Resistant Hospital-Acquired Isolates. Int J Mol Sci.

[35] Emelyanenko AM, Omran FS, Teplonogova MA, Chernukha MY, Avetisyan LR, Tselikina EG, Putsman GA, Zyryanov SK, Butranova OI, Emelyanenko KA, Boinovich LB (2024). An Antimicrobial Copper-Plastic Composite Coating: Characterization and In Situ Study in a Hospital Environment. Int J Mol Sci.

[36] Aillón-García P, Parga-Landa B, Guillén-Grima F (2023). Effectiveness of copper as a preventive tool in health care facilities. A systematic review. Am J Infect Control.

[37] Zhao C, Zhou L, Chiao M, Yang W (2020). Antibacterial hydrogel coating: Strategies in surface chemistry. Adv Colloid Interface Sci.

[38] Yan Z, Yao M, Zhao Z, Yang Q, Liu R, Liu B, Wang X, Chen L, Zhang H, Wei Y, Yao F, Li J (2024). Mechanical-Enhanced and Durable Zwitterionic Hydrogel Coating for Inhibiting Coagulation and Reducing Bacterial Infection. Adv Healthc Mater.

[39] Mikolay A, Huggett S, Tikana L, Grass G, Braun J, Nies DH (2010). Survival of bacteria on metallic copper surfaces in a hospital trial. Appl Microbiol Biotechnol.

[40] Poole K (2017). At the Nexus of Antibiotics and Metals: The Impact of Cu and Zn on Antibiotic Activity and Resistance. Trends Microbiol.

[41] Yu Z, Gunn L, Wall P, Fanning S (2017). Antimicrobial resistance and its association with tolerance to heavy metals in agriculture production. Food Microbiol.

[42] Singh CK, Sodhi KK, Shree P, Nitin V (2024). Heavy Metals as Catalysts in the Evolution of Antimicrobial Resistance and the Mechanisms Underpinning Co-selection. Curr Microbiol.

[43] Li X, Rensing C, Vestergaard G, Arumugam M, Nesme J, Gupta S, Brejnrod AD, Sørensen SJ (2022). Metagenomic evidence for co-occurrence of antibiotic, biocide and metal resistance genes in pigs. Environ Int.

[44] Rensing C, Moodley A, Cavaco LM, McDevitt SF (2018). Resistance to Metals Used in Agricultural Production. Microbiol Spectr.

[45] Argudín MA, Hoefer A, Butaye P (2019). Heavy metal resistance in bacteria from animals. Res Vet Sci.

[46] Cidre I, Pulido RP, Burgos MJG, Gálvez A, Lucas R (2017). Copper and Zinc Tolerance in Bacteria Isolated from Fresh Produce. J Food Prot.

[47] Yazdankhah S, Rudi K, Bernhoft A (2014). Zinc and copper in animal feed - development of resistance and co-resistance to antimicrobial agents in bacteria of animal origin. Microb Ecol Health Dis.

[48] van Alen S, Kaspar U, Idelevich EA, Köck R, Becker K (2018). Increase of zinc resistance in German human derived livestock-associated MRSA between 2000 and 2014. Vet Microbiol.

[49] Zhong Q, Pan X, Chen Y, Lian Q, Gao J, Xu Y, Wang J, Shi Z, Cheng H (2024). Prosthetic Metals: Release, Metabolism and Toxicity. Int J Nanomedicine.

[50] Heiden SE, Hübner NO, Bohnert JA, Heidecke CD, Kramer A, Balau V, Gierer W, Schaefer S, Eckmanns T, Gatermann S, Eger E, Guenther S, Becker K, Schaufler K (2020). A Klebsiella pneumoniae ST307 outbreak clone from Germany demonstrates features of extensive drug resistance, hypermucoviscosity, and enhanced iron acquisition. Genome Med.

[51] Facciolà A, Visalli G, La Maestra S, Ceccarelli M, D'Aleo F, Nunnari G, Pellicanò GF, Di Pietro A (2019). Carbon nanotubes and central nervous system: Environmental risks, toxicological aspects and future perspectives. Environ Toxicol Pharmacol.

[52] Wojgani H, Kehsa C, Cloutman-Green E, Gray C, Gant V, Klein N (2012). Hospital door handle design and their contamination with bacteria: a real life observational study. Are we pulling against closed doors?. PLoS One.

[53] Preiss S, Kramer A (2011). Foot-operated door opener to eliminate the door handle as a source of contamination. GMS Krankenhhyg Interdiszip.

[54] Schweizer ML, Reisinger HS, Ohl M, Formanek MB, Blevins A, Ward MA, Perencevich EN (2014). Searching for an optimal hand hygiene bundle: a meta-analysis. Clin Infect Dis.

[55] Aboelela SW, Stone PW, Larson EL (2007). Effectiveness of bundled behavioural interventions to control healthcare-associated infections: a systematic review of the literature. J Hosp Infect.

